# Postoperative pulmonary complications after sugammadex reversal of neuromuscular blockade: a systematic review and meta-analysis with trial sequential analysis

**DOI:** 10.1186/s12871-023-02094-0

**Published:** 2023-04-20

**Authors:** Hong-Mei Liu, Hong Yu, Yi-Ding Zuo, Peng Liang

**Affiliations:** 1 Department of Anesthesiology, West China Hospital, Sichuan University, No.37 Guoxue Alley, Sichuan, Chengdu 610041 China; 2 Day Surgery Center, West China Hospital, Sichuan University, Chengdu, 610041 China

**Keywords:** Neuromuscular blocking agents, Postoperative pulmonary complications, Sugammadex, Neostigmine, Meta-analysis

## Abstract

**Background:**

Sugammadex has been reported to lower the incidence of postoperative residual neuromuscular blockade. Despite the advantages, until recently the effects of sugammadex on postoperative pulmonary complications (PPCs) were controversial. We conducted a systematic review and meta-analysis to determine whether reversal with sugammadex was associated with a lower risk of PPCs compared with neostigmine.

**Methods:**

PubMed, Embase, and Cochrane Central Register of Controlled Trials were searched from inception to May 2022. Randomized controlled trials (RCTs) and observational studies comparing PPCs in patients receiving sugammadex or neostigmine as reversal agent at the end of surgery were included. The primary outcomes focused on PPCs including desaturation, pneumonia, atelectasis, noninvasive ventilation (NIV) and reintubation. Trial sequential analysis was performed on the primary outcomes to confirm whether firm evidence was reached.

**Results:**

Meta-analysis of included studies showed that the rate of desaturation (43.2% vs 45.0%, RR = 0.82; 95% CI 0.63 to 1.05; *p* = 0.11) were comparable between the two groups. When looking at other primary outcomes, significantly lower risk of pneumonia (1.37% vs 2.45%, RR = 0.65; 95% CI 0.49 to 0.85; *p *= 0.002), atelectasis (24.6% vs 30.4%, RR = 0.64; 95% CI 0.42 to 0.98; *p* = 0.04), NIV (1.37% vs 2.33%, RR = 0.65; 95% CI 0.43 to 0.98; *p* = 0.04) and reintubation (0.99% vs 1.65%, RR = 0.62; 95% CI 0.43 to 0.91; *p* = 0.01) in the sugammadex group were detected compared with the neostigmine group.

**Conclusions:**

We concluded that sugammadex is more effective at reducing the incidence of PPCs including pneumonia, atelectasis, NIV and reintubation compared with neostigmine. Further evidence, preferably from RCTs, is required to confirm these findings.

**Supplementary Information:**

The online version contains supplementary material available at 10.1186/s12871-023-02094-0.

## Background

The use of neuromuscular blocking drugs is considered essential during general anesthesia, but may contribute to residual neuromuscular blockade (NMB) [1]. Numerous studies have shown that residual NMB was associated with impaired upper airway patency, decreased functional residual capacity, and respiratory insufficiency, consequently putting patients at risk of multiple postoperative pulmonary complications (PPCs) [2, 3]. Therefore, full restoration of muscle strength may decrease the risk of PPCs, including hypoxemia, atelectasis, and pneumonia [4, 5].

Acetylcholinesterase (AChE) inhibitors like neostigmine are used to reduce residual NMB through increasing the amount of acetylcholine in the synaptic cleft. However, the application of neostigmine cannot always ensure complete restoration of patients’ muscle strength. In addition, routine reversal with neostigmine at the end of the surgery may not only cause a variety of side effects (e.g. bradycardia, bronchoconstriction, hypersalivation), but also adversely affect neuromuscular functions [6, 7].

Meanwhile, sugammadex, a novel reversal agent, has been reported to lower the incidence of postoperative residual NMB with more rapid reversal [8]. Besides, sugammadex has no endogenous targets, so no major adverse effects will be caused [8]. Despite the advantages, until recently the effects of sugammadex on PPCs were controversial [9–15]. Therefore, in order to address this question, we performed this systematic review and meta-analysis of current evidence to evaluate whether reversal with sugammadex was associated with a lower risk of postoperative PPCs compared with reversal with neostigmine.

## Methods

We followed the Cochrane Handbook for Systematic Reviews of Interventions statement and the Preferred Reporting Items for Systematic Reviews and Meta-Analyses statement (PRISMA) (Supplementary file 1). The protocol for this review was registered on PROSPERO (CRD42021253820) on June 7, 2021. Ethical approval and patient consent are not required in a meta-analysis.

### Search strategy

Systematic research was performed on PubMed, EMBASE, and Cochrane Central Register of Controlled Trials (CENTRAL) with retrieval time from inception to May 2022. The search was restricted to articles published in English language and full-text versions. Our search strategy was based on two search themes, using: 1) “Sugammadex” or “selective relaxant binding agent of SRBA” or “org 25,969” or “bridion” and 2) “pulmonary complications” or “pneumonia” or “respiratory complications” (Supplementary file 2). A manual search was also conducted to identify additional relevant studies.

### Selection criteria

Inclusion criteria were as follows: 1) design: randomized controlled trials (RCTs) or observational studies; 2) population: adult patients (> 18 years) who received non-depolarizing neuromuscular blocking agents for surgery; 3) intervention: receiving sugammadex reversal of neuromuscular blockade; 4) control: receiving neostigmine reversal of neuromuscular blockade. The dose of sugammadex and neostigmine, and the time-point of administration of the study drug were not limited; 5) outcomes: eligible studies must report at least one type of the pulmonary or respiratory complications. The primary outcomes focused on PPCs including desaturation, pneumonia, atelectasis, noninvasive ventilation (NIV) and reintubation. Secondary outcomes were other PPCs including pleural effusion, aspiration pneumonia, airway obstruction and pneumothorax.

### Data extraction

Two investigators independently screened the titles and abstracts of initial search results with compliance to selection criteria and selected studies for the final analysis. Data were extracted with a standard form. The collected information was as follows: first author, year of publication, study design, sample size, surgical procedure, dose of study drugs. Divergences were finally resolved by consensus with the corresponding author.

### Validity assessment

The quality of RCTs was assessed by the Cochrane Collaboration risk of bias tool in seven aspects: random sequence generation, allocation concealment, blinding of participants and personnel, blinding of outcome assessment, incomplete outcome data, selective reporting and other biases. Observational studies were evaluated according to the Newcastle–Ottawa scale (NOS) that contained three parts: patient selections, comparability of the study groups, and assessment of outcomes. The score of each part was 4, 2, and 3, respectively. A high-quality study was defined as an overall quality score ≥ 7 [16].

### Quality of evidence

Quality of evidence was evaluated by GRADE (Grades of Recommendation, Assessment, Development, and Evaluation) system using the Guideline Development Tool ( https://www.grade.pro.org).

### Statistical analysis

All analyses were performed using computer programs including Review Manager (RevMan) V.5.4. For primary and secondary outcomes, we estimated the risk ratio (RR) with 95% confidence intervals (CIs) using the random effects model. The I^2^ statistics was used to assess the studies’ heterogeneity. I^2^ values of 0% to 24.9%, 25% to 49.9%, 50% to 74.9%, and 75% to 100% indicated none, low, moderate, and high thresholds for statistical heterogeneity. Furthermore, we performed subgroup analyses according to study type (RCTs and observational studies) and sensitivity analyses to explore the sources of heterogeneity. A funnel plot was used to estimate potential publication bias for analyses over 10 studies. A pre-specified trial sequential analysis (TSA) was performed on the primary outcomes using TSA software (Copenhagen Trial Unit, Center for Clinical Intervention Research, Copenhagen). Statistical significance was set at a two-sided *P* value < 0.05.

## Results

### Literature identification and study characteristics

**Fig. 1:**
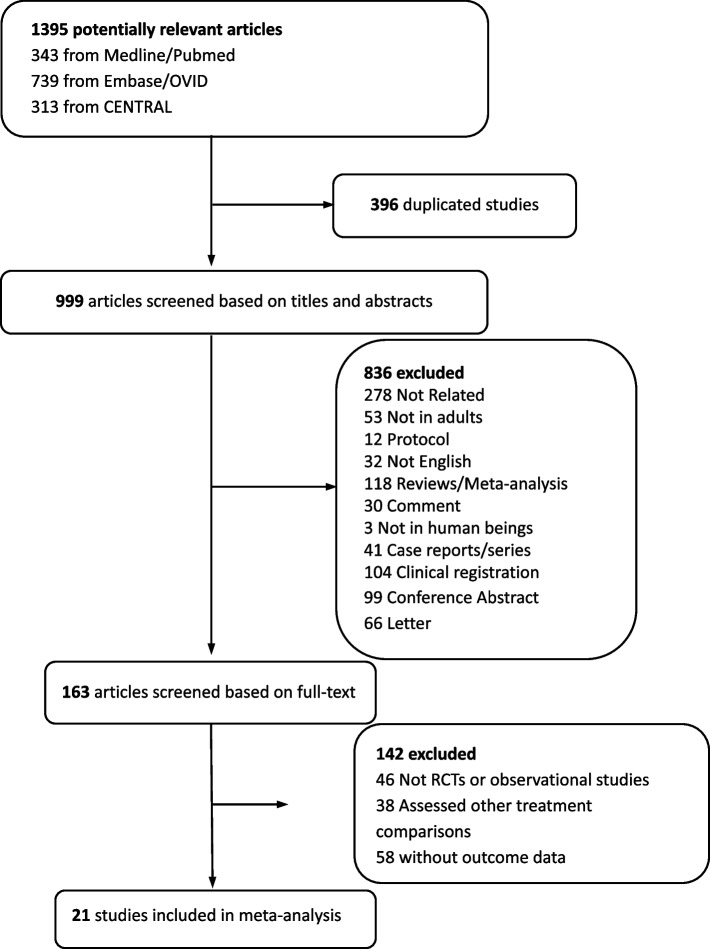
Flow chart of selecting process in this meta-analysis

We initially identified 1395 potentially eligible articles in the database searches (343 from Medline/PubMed, 739 from Embase/OVID and 313 from CENTRAL). The results are summarized in the PRISMA diagram (Fig. 1). Eventually, 21 studies were included based on the full text. The characteristics of the eligible studies were described in Table 1.

**Table 1:** Characteristics of included studies

		**Sample size**			**Doses**		**NOS**
**Study**	**Design**	**sugammadex**	**neostigmine**	**Surgical procedure**	**sugammadex**	**neostigmine**	**selection/comparability/outcome**
Ledowski (2013) [24]	SC, POS	57	89	No restriction	110-400 mg	1.25–5 mg	4/1/3
Llauradó (2014) [25]	SC, POS	160	160	laparoscopic bariatric surgery	2 or 4 mg/kg	0.04 mg/kg	4/1/3
Ezri (2015) [15]	SC, ROS	112	67	Laparoscopic sleeve gastrectomy	1.5-2 mg/kg	2.5 mg	4/1/3
Ünal (2015) [14]	SC, RCT	37	37	Treatment of OSA	2 mg/kg	0.04 mg/kg	/
Evron (2017) [17]	SC, RCT	32	25	laparoscopic sleeve gastrectomy	2 mg/kg	2.5 mg	/
Alday (2019) [13]	SC, RCT	62	64	Major abdominal surgery	4 mg/kg	0.04 mg/kg	/
Çitil (2019)	SC, RCT	30	30	Lung Resection Surgery	2 mg/kg	0.05 mg/kg	/
Han^a ^(2020) [11]	SC, ROS/PS	616	616	Laparoscopic sleeve gastrectomy	2 or 4 mg/kg	0.02–0.05 mg/kg	4/2/3
Kheterpal^a ^(2020) [10]	MC, ROS/PS	22,856	22,856	No restriction	1.8–4.4 mg/kg	NR	4/2/3
Krause^a ^(2020) [12]	SC, ROS/PS	3896	3420	No restriction	NR	NR	4/2/3
Lee (2020) [19]	SC, RCT	37	36	Laparoscopic cholecystectomy	2-4 mg/kg	0.02–0.05 mg/kg	/
Moon (2020) [20]	SC, RCT	44	48	Thoracic operation	2 mg/kg	0.05 mg/kg	/
Togioka (2020) [9]	SC, RCT	100	100	No restriction	2 mg/kg	0.07 mg/kg	/
Goodner (2021) [29]	SC,ROS	117	154	No restriction	2.05 ± 0.92 mg/kg	0.042 ± 0.011 mg/kg	4/1/3
Ledowski (2021) [37]	MC,RCT	85	83	No restriction	2 mg/kg	0.05 mg/kg	/
Lee (2021) [21]	SC, RCT	46	47	VATS for lobectomy	2 mg/kg	0.05 mg/kg	/
Leslie (2021) [23]	MC,RCT	59	61	Abdominal, retroperitoneal, pelvic and non-cardiac intrathoracic surgery	NR	NR	/
Li (2021) [26]	SC, ROS	2691	7800	No restriction	NR	NR	4/1/3
Murphy (2021) [30]	SC,POS	97	100	Thoracoscopic surgery	4 mg/kg	0.07 mg/kg	4/2/3
Yu^a ^(2021) [27]	SC, ROS/PS	237	237	RALP	2 mg/kg	0.04 mg/kg	4/2/3
Cheng (2022) [28]	SC, ROS	215	118	da Vinci surgery	2 or 4 mg/kg	0.02–0.04 mg/kg	4/1/3

*SC* Single center, *MC* Multiple center, *POS* Prospective observational study, *ROS* Retrospective observational study, *RCT* Randomized controlled trial, *PS* Propensity score matching, *OSA* Obstructive sleep apnea, *VATS* Video-assisted thoracoscopic surgery, *RALP* Robot-assisted laparoscopic prostatectomy, *NOS* Newcastle–Ottawa Quality Assessment Scale-Cohort Studies, *NR* Not reported

^a^Sample size after propensity score matching

Ten RCTs [9, 13, 14, 17–23] (1123 patients) and eleven observational studies [10–12, 15, 24–30] (66,671 patients) were identified for final analyses. The sample size of the included studies ranged from 57 to 45,712 adults. Three studies were multi-centered [10, 22, 23], whereas the rest had a single-center design. The patients in the included studies underwent various surgeries. Seven studies were performed with no surgical procedure restriction [9, 10, 12, 22, 24, 29, 31], four studies on patients undergoing laparoscopic sleeve gastrectomy [11, 15, 17, 25], four on thoracic surgery [18, 20, 21, 30]. Other procedures included laparoscopic cholecystectomy[19], robot-assisted laparoscopic prostatectomy [27], major abdominal surgery[13, 23], da Vinci surgery [28] and treatment of obstructive sleep apnea [14].

Additionally, although all studies compared sugammadex and neostigmine, the drugs were administered in different doses. The doses of sugammadex ranged from 1.5 to 4 mg/kg and the doses of neostigmine ranged from 0.02–0.07 mg/kg. All but three studies [11, 23, 26] reported the quantitative neuromuscular monitoring using the train-of-four ratio with a nerve stimulator.

### Quality assessment

**Fig. 2:**
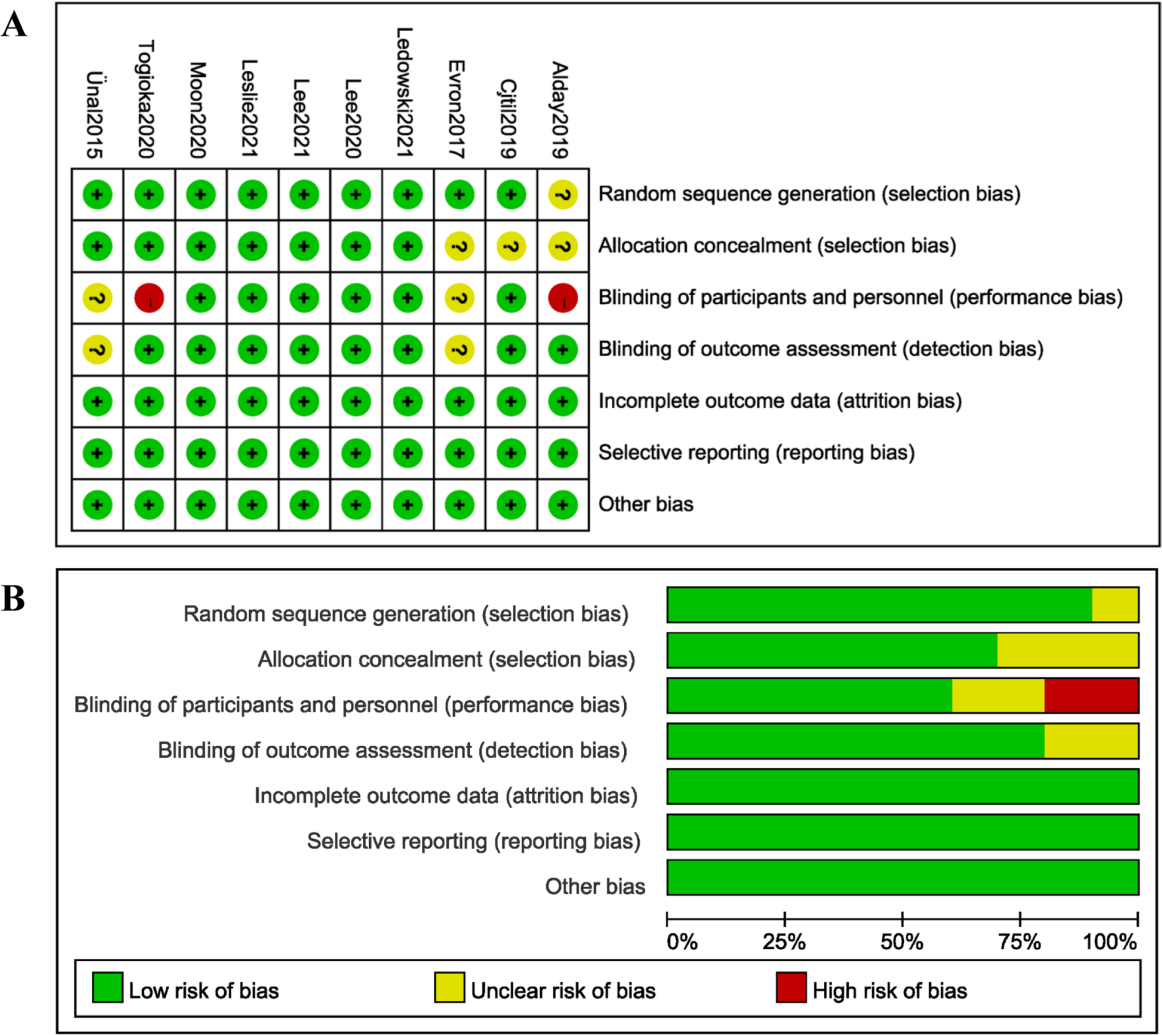
Assessment of risk bias: (**A**) a summary of bias for each included RCT study; (**B**) a graph with percentages for all included RCT studies

Quality assessments of included RCTs were shown in Fig. 2 and observational studies were shown in Table 1. Study quality appraisal showed that 7 of 10 RCTs [9, 14, 19–23] described appropriate methods used for randomized sequence generation and allocation concealment and 6 of 10 RCTs [18–23] performed the blinding of both participants and personnel and outcome assessment. 5 RCTs [19–23] were of high quality, whereas others lacked important details to appraise the risk of selection, performance, attrition, or detection biases. Quality assessments of observational studies showed all of them were ranked as publications with high quality with a score of 8 or 9.

### Primary outcomes

**Fig. 3:**
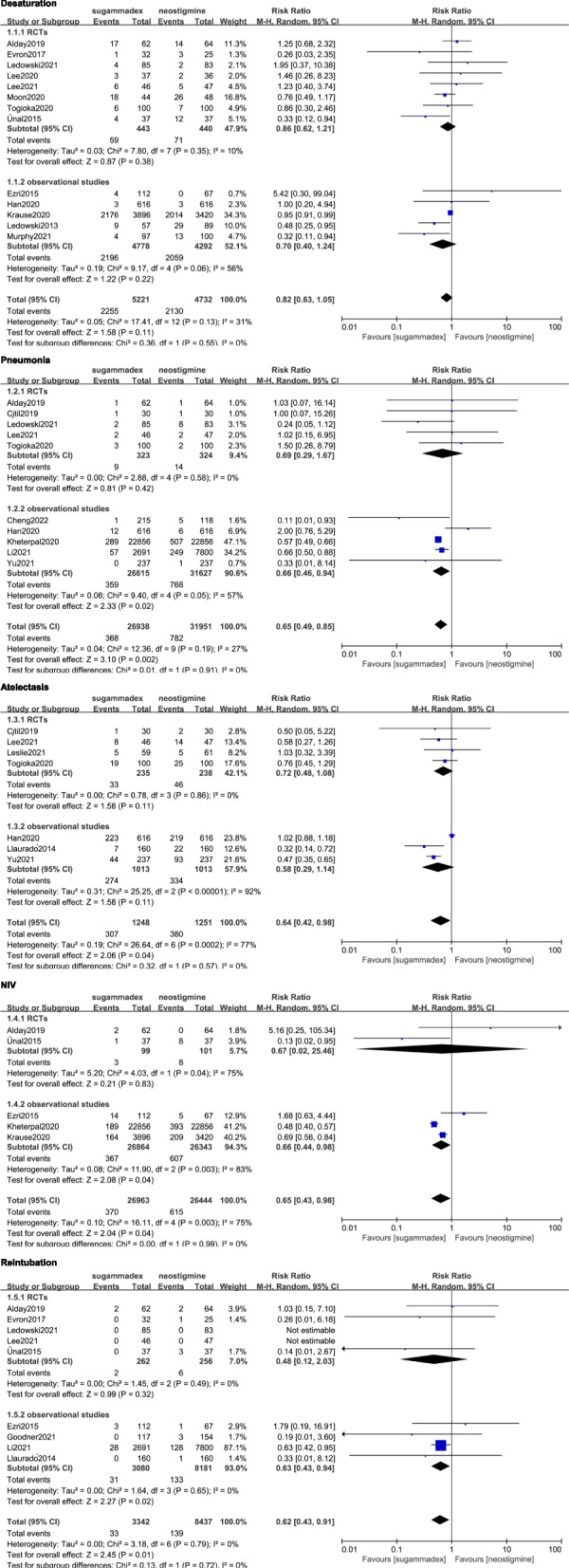
Forest plot showing the comparison of primary outcomes between the sugammadex and neostigmine. A pooled RR was calculated using the random-effect model according to the Mantel–Haenszel (M-H) method. RCT, randomized controlled trials; NIV, noninvasive ventilation

Meta-analysis of 8 RCTs [9, 13, 14, 17, 19–22] and 5 observational studies [11, 12, 15, 24, 30] showed that the rate of desaturation (43.2% vs 45.0%, RR = 0.82; 95% CI 0.63 to 1.05; *p* = 0.11; p for heterogeneity = 0.13, I^2^ = 31%; Fig. 3, publication bias in Supplementary file 3) were comparable between the two groups.

When looking at other primary outcomes, a significantly lower risk of the following PPCs in the sugammadex were detected when compared with neostigmine: pneumonia (1.37% vs 2.45%, RR = 0.65; 95% CI 0.49 to 0.85; *p* = 0.002; p for heterogeneity = 0.19, I^2^ = 27%; Fig. 3, publication bias in Supplementary file 3) by pooling results from 5 RCTs [9, 13, 18, 21, 22] and 5 observational studies [10, 11, 26–28]; atelectasis (24.6% vs 30.4%, RR = 0.64; 95% CI 0.42 to 0.98; *p* = 0.04; p for heterogeneity = 0.0002, I^2^ = 77%; Fig. 3) from 4 RCTs [9, 18, 21, 23] and 3 observational studies [11, 25, 27]; NIV (1.37% vs 2.33%, RR = 0.65; 95% CI 0.43 to 0.98; *p* = 0.04; p for heterogeneity = 0.003, I^2^ = 75%; Fig. 3) from 2 RCTs [13, 14] and 3 observational studies [10, 12, 15]; and reintubation (0.99% vs 1.65%, RR = 0.62; 95% CI 0.43 to 0.91; *p* = 0.01; p for heterogeneity = 0.79, I^2^ = 0%; Fig. 3) from 5 RCTs [13, 14, 17, 21, 22] and 4 observational studies [15, 25, 26, 29].

### Secondary outcomes

**Fig. 4:**
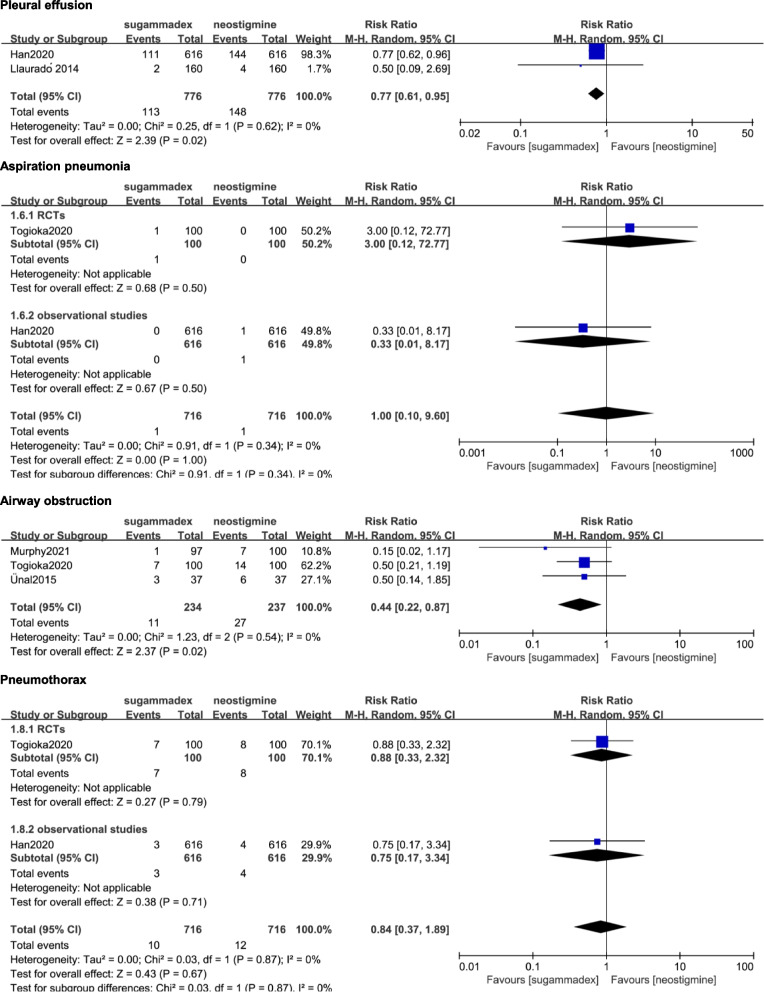
Forest plot showing the comparison of secondary outcomes between the sugammadex and neostigmine. A pooled RR was calculated using the random-effect model according to the Mantel–Haenszel (M-H) method. RCT, randomized controlled trials

Pooled data showed a significant lower rate of pleural effusion (14.6% vs 19.1%, RR = 0.77; 95% CI 0.61 to 0.95; *p* = 0.02; p for heterogeneity = 0.62, I^2^ = 0%; Fig. 4) from two observational studies [11, 25] and airway obstruction (4.7% vs 11.4%, RR = 0.44; 95% CI 0.22 to 0.87; *p* = 0.02; p for heterogeneity = 0.54, I^2^ = 0%; Fig. 4) from three observational studies [9, 14, 30] in the sugammadex group compared with that in the neostigmine group.

There were no significant reductions with the use of sugammadex compared to neostigmine in aspiration pneumonia (0.14% vs 0.14%, RR = 1.00; 95% CI 0.10 to 9.60; *p* = 1.00; p for heterogeneity = 0.34, I^2^ = 0%; Fig. 4) from one RCT [9] and one observational study [11] and pneumothorax (1.40% vs 1.68%, RR = 0.84; 95% CI 0.37 to 1.89; *p* = 0.67; p for heterogeneity = 0.87, I^2^ = 0%; Fig. 4) from one RCT [9] and one observational study [11].

### Sensitivity analyses

Sensitivity analyses were performed according to study design (RCTs and observational studies) (Fig. 3 and Fig. 4). The pooled data of the desaturation, pneumonia, NIV and reintubation from observational studies had no change. However, the pooled data of all primary outcomes from RCTs showed no significance between the two groups.

### Quality of evidence

GRADE system grades of evidence are low certainty for desaturation, pneumonia, and very low certainty for atelectasis, NIV and reintubation (Supplementary file 4).

### Trial sequential analysis

The cumulative Z-score curve (blue line) of desaturation neither crossed the traditional significance boundary nor reached the required information size, which indicated that more trials were needed to detect the effect of sugammadex on the incidence of desaturation compared to neostigmine. The Z-score curves (blue line) of pneumonia and NIV crossed both the required information size (RIS) (vertical red line) and the conventional statistical significance boundary (dotted red lines), which indicated that the observed reduction in rate of pneumonia and NIV in patients using sugammadex could be considered conclusive with the existing evidence. The Z-score curve (blue line) of atelectasis and reintubation crossed the conventional statistical significance boundary (dotted red lines), but did not reach the RIS, which indicated that more trials were needed to reliably detect a plausible effect of sugammadex on the incidence of atelectasis and reintubation (Supplementary file 5). TSA for secondary outcomes were ignored due to too little information used to calculate RIS.

## Discussion

To the best of our knowledge, this is the first systematic review and meta-analysis to collect all available data from clinical trials to find out whether reversal with sugammadex was associated with a lower risk of PPCs compared with reversal with neostigmine. In the current study, we performed a comprehensive search with broad search terms, without limiting the search to RCTs or by surgical type.

The major findings of our study indicated that the use of sugammadex as a reversal agent compared to neostigmine decreased the incidence of pneumonia, atelectasis, NIV, reintubation, pleural effusion and airway obstruction. However, the incidence of desaturation, aspiration pneumonia and pneumothorax did not differ significantly between the two groups.

The use of intermediate acting NMB agents during anesthesia was associated with an increased risk of clinically meaningful respiratory complications [7]. However, neostigmine, a commonly used AChE inhibitors, administered after full recovery of neuromuscular function may result in paradoxical muscle weakness [32]. However, sugammadex, a novel reversal agent, acts 10 times more rapidly than neostigmine, which indicates that it can reverse neuromuscular blockade more rapidly and completely than neostigmine and lower the incidence of residual paralysis [8, 33]. Therefore, sugammadex was considered to have the potential to reduce PPCs compared to neostigmine.

Previous meta-analysis [34] comparing sugammadex with neostigmine in older patients found that the application of sugammadex was associated with lower incidence of pneumonia, which was consistent with our study. However, there existed differences between that study and our meta-analysis. First, that study included only three RCTs (*n* = 510), introducing risk of bias. Second, previous studies showed that sugammadex was related to rapid and complete restoration of muscle strength [8, 33, 35], improving muscle tone of upper airway and chest wall, thus enabling patients to cough, decreased alveolar collapse and prevented microaspiration [36]. Therefore, older patients with worse metabolic function may benefit more from sugammadex [9, 37], which suggested that the conclusion of Carron’s study [34] was less generalizable than our meta-analysis.

In the current meta-analysis, the pooled data of pneumonia, atelectasis, NIV and reintubation all favored sugammadex. Application of TSA indicated that we can draw a confirm conclusion that sugammadex appears superior to neostigmine in decreasing the risk of pneumonia and NIV. These studied outcomes, pneumonia, NIV and reintubation, could represent reliable and impactful pulmonary complications, unlike less severe but more frequent events such as desaturation, and atelectasis [38]. What is noteworthy of this finding is that the superiority of sugammadex might have been largely influenced by the results from Kheterpal et al. (a multicenter matched cohort study including 45,712 participants) as they found sugammadex was associated with a 47% reduced risk of pneumonia, and a 55% reduced risk of respiratory failure compared to neostigmine [10].

Our secondary outcomes, which included pleural effusion, aspiration pneumonia, airway obstruction and pneumothorax, were also likely to be related to neuromuscular blockade. However, these pulmonary outcomes were poorly described in our included studies and a firm conclusion could not be drawn.

There are several potential limitations as our findings are limited by the quality and quantity of available evidence in the included trials. First, according to the GRADE system, the certainty of our findings ranked very low to low across different outcomes. The main limiting factors that contribute to the low overall quality included the high risk of bias and observational study design of included studies. Second, some of the included studies poorly described the outcomes of interest (such as PPCs defined as secondary outcomes). Third, the sample size of included RCTs was limited. Therefore, the findings might be largely influenced by the observational studies. Fourth, the study design of included studies varied in surgical type, time and dosage of drug administration and definition of PPCs, but we pooled data of individual pulmonary complications. Also, the calculated heterogeneity related to the clinical outcomes was very low and sensitivity analyses were also performed. Further evidence, preferably from RCTs, is required to confirm our findings. Fifth, neuromuscular monitoring is strongly recommended whenever NMB agents are administered to decrease the risk of PPCs [39]. However, among 21 studies, 17 studies applied neuromuscular monitoring. And the population who was not monitored may have higher incidence of PPCs, which would obfuscate the results. Furthermore, we did not test for publication bias with datasets of less than 10 data, so publication bias could not be excluded for some outcomes.

## Conclusions

The results from this systematic review and meta-analysis suggested that reversal of neuromuscular block with sugammadex decreased the incidence of PPCs including pneumonia, atelectasis, NIV, reintubation, pleural effusion and airway obstruction. The evidence was not strong enough to draw firm conclusions about other PPCs including desaturation, aspiration pneumonia and pneumothorax. More sufficiently powered, prospective randomized studies are warranted to confirm this effect size.

## Supplementary Information

**Additional file 1:  Supplementary file 1. **PRISMA Checklist.

**Additional file 2: Supplementary file 2. **Electronic search strategies.

**Additional file 3: Supplementary file 3. **Funnel plot.

**Additional file 4: Supplementary file 4. **Quality of evidence by GRADE.

**Additional file 5: Supplementary file 5. **TSA.

## Data Availability

The datasets used and/or analysed during the current study available from the corresponding author on reasonable request.

## Abbreviations

AChE: Acetylcholinesterase
CENTRAL: Cochrane Central Register of Controlled Trials
CIs: Confidence intervals
GRADE: Grades of Recommendation, Assessment, Development, and Evaluation
NIV: Noninvasive ventilation
NMB: Neuromuscular blockade
NOS: Newcastle–Ottawa scale
PPCs: Postoperative pulmonary complications
PRISMA: Preferred Reporting Items for Systematic Reviews and Meta-Analyses
RCTs: Randomized controlled trials
RIS: Required information size
RR: Risk ratio
TSA: Trial sequential analysis
