# Supplementary material for: Postoperative pulmonary complications after sugammadex reversal of neuromuscular blockade: a systematic review and meta-analysis with trial sequential analysis
Source: BMC Anesthesiol. 2023 Apr 20;23:130. doi: 10.1186/s12871-023-02094-0 (PMC10116764; doi:10.1186/s12871-023-02094-0)
Supplement: Supplementary file 2 — Additional file 2: Supplementary file 2. Electronic search strategies. [file 12871_2023_2094_MOESM2_ESM.docx]

**Supplementary file 2: Electronic search strategies**

**Medline/Pubmed**

1. Sugammadex
2. selective relaxant binding agent
3. SRBA
4. org 25969
5. bridion
6. **1 or 2 or 3 or 4 or 5**
7. neostigmine
8. **6 and 7**

**Embase/Ovid**

1. Sugammadex
2. selective relaxant binding agent
3. SRBA
4. org 25969
5. bridion
6. **1 or 2 or 3 or 4 or 5**
7. neostigmine
8. **6 and 7**

**Cochrane library**

1. Sugammadex
2. selective relaxant binding agent
3. SRBA
4. org 25969
5. bridion
6. **1 or 2 or 3 or 4 or 5**
7. neostigmine
8. **6 and 7**
