# Supplementary figures and images for: Postoperative pulmonary complications after sugammadex reversal of neuromuscular blockade: a systematic review and meta-analysis with trial sequential analysis
Source: BMC Anesthesiol. 2023 Apr 20;23:130. doi: 10.1186/s12871-023-02094-0 (PMC10116764; doi:10.1186/s12871-023-02094-0)

Supplementary file 3: Funnel plot

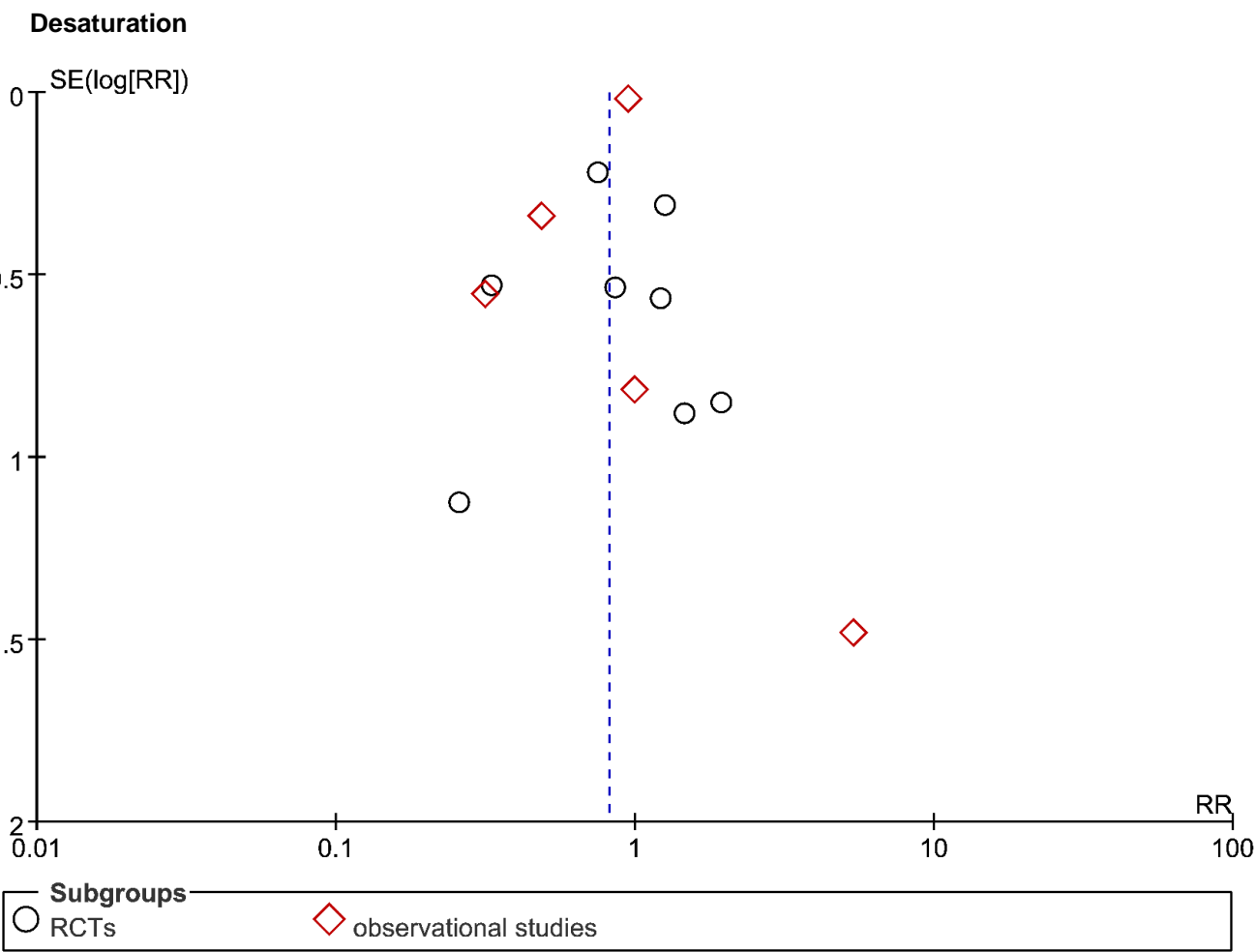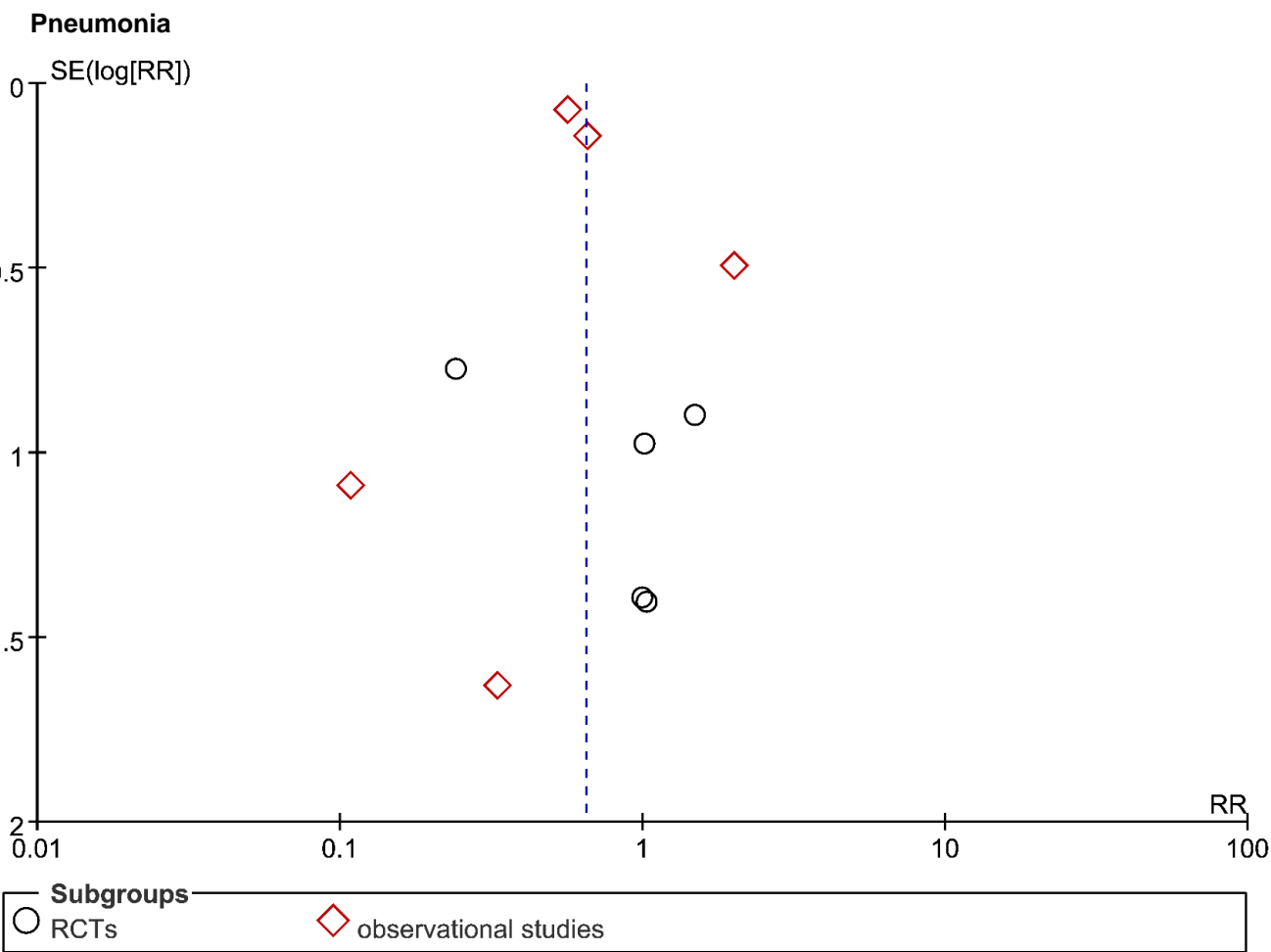

Supplement: Supplementary file 3 — Additional file 3: Supplementary file 3. Funnel plot. [file 12871_2023_2094_MOESM3_ESM.pdf]

Supplementary file 5: TSA

Desaturation

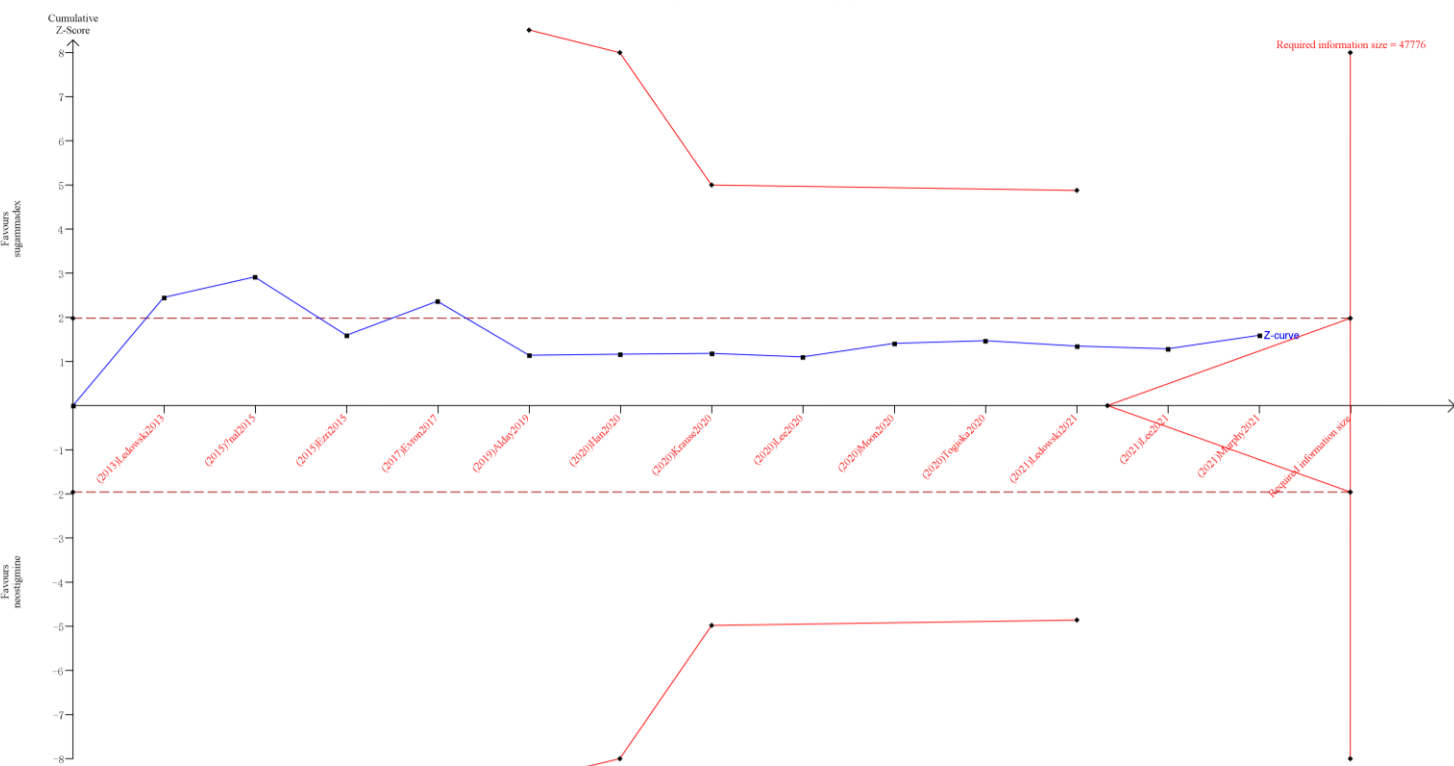

Pneumonia

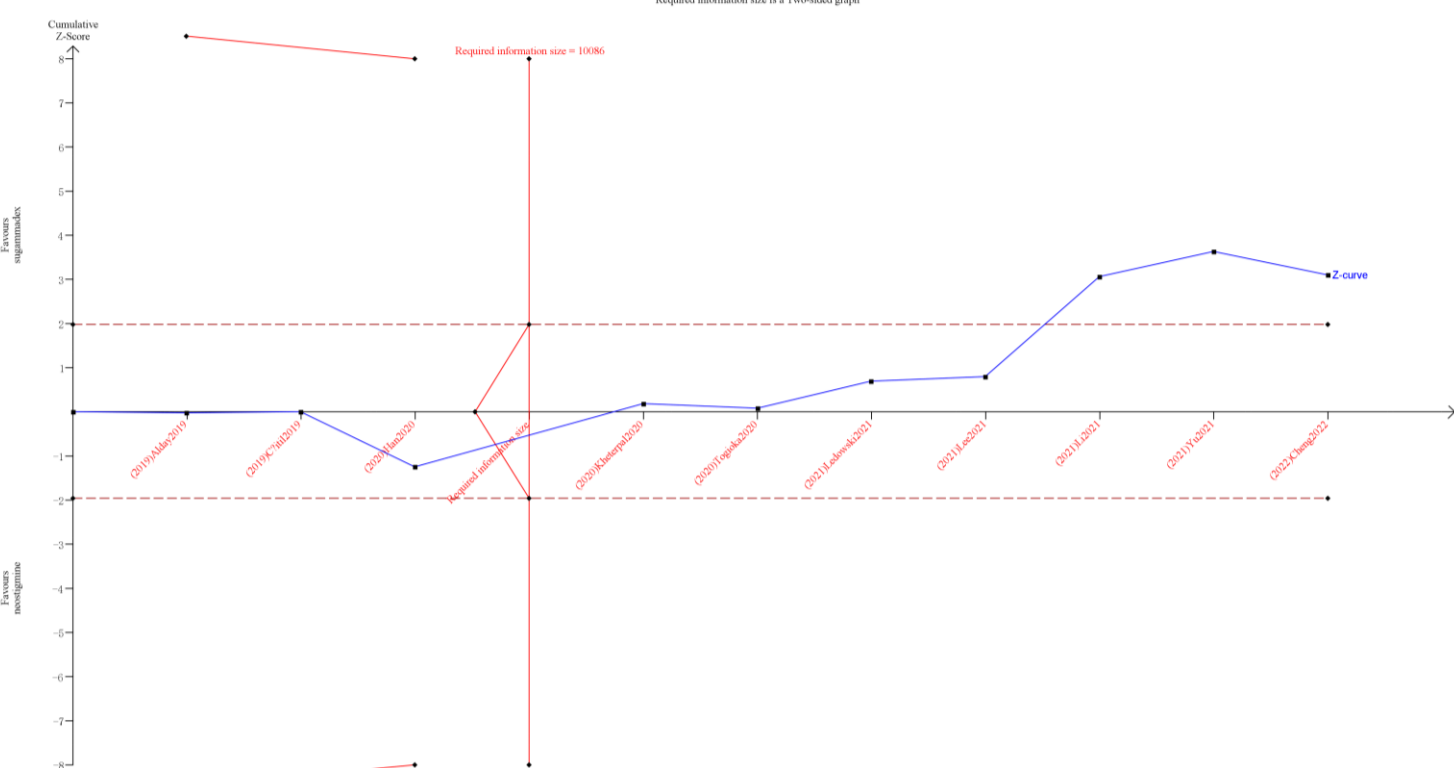

Atelectasis

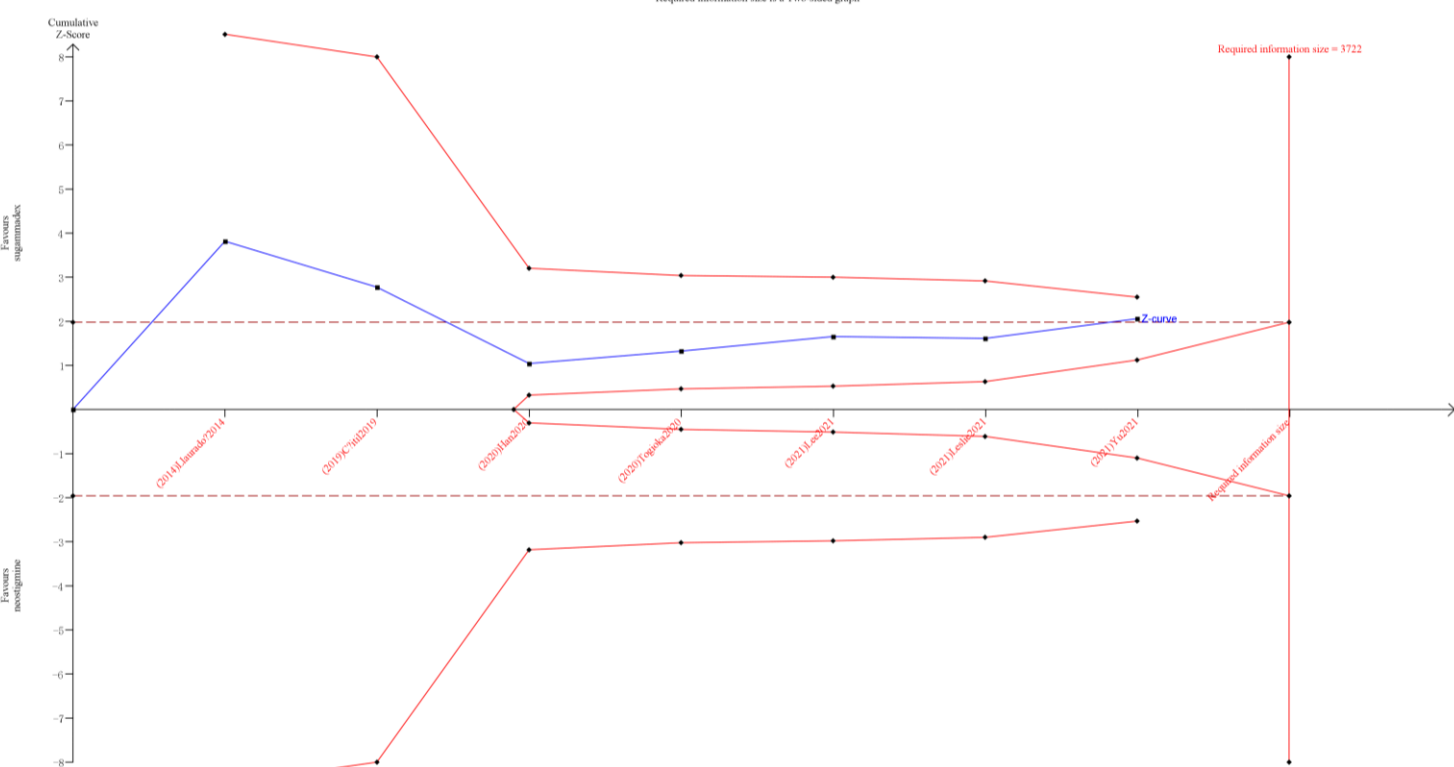

NIV

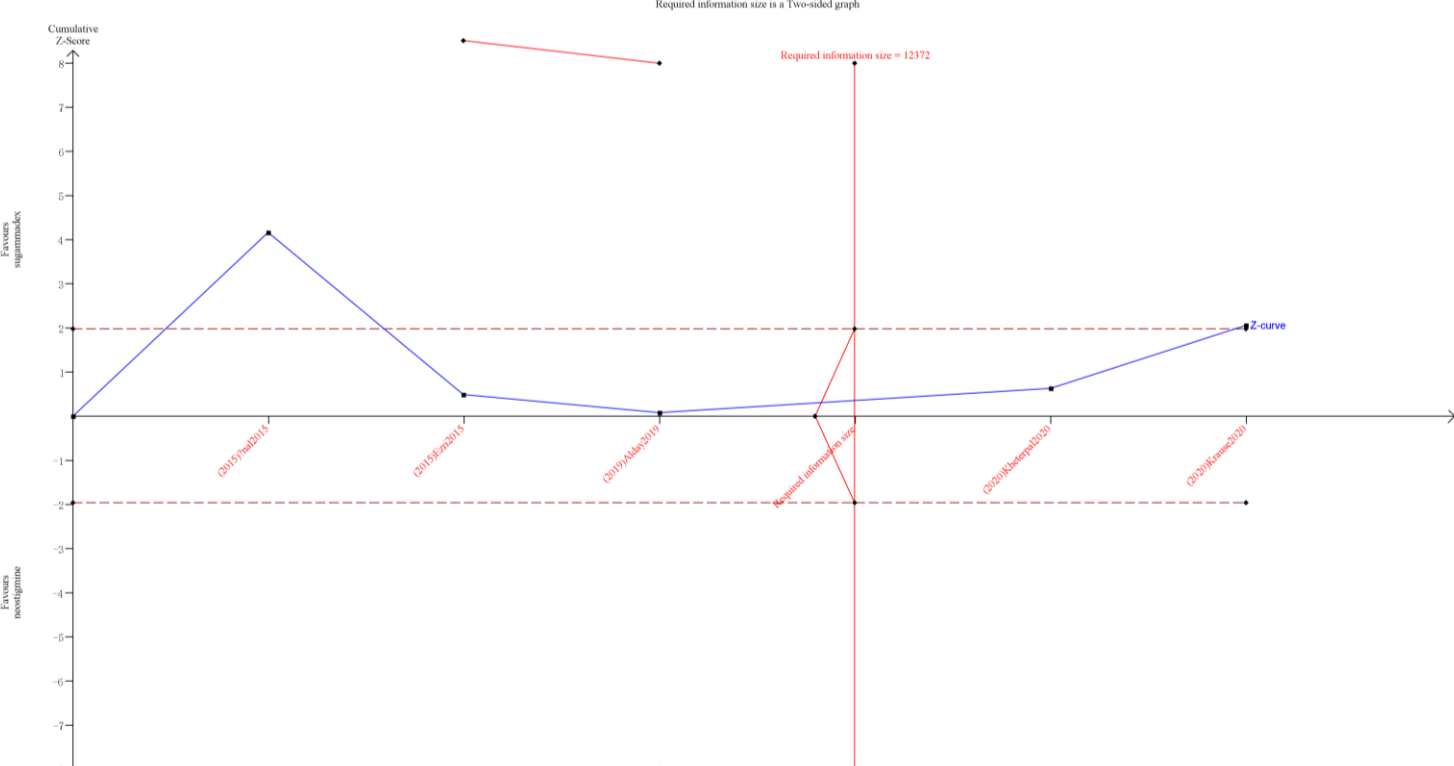

Reintubation

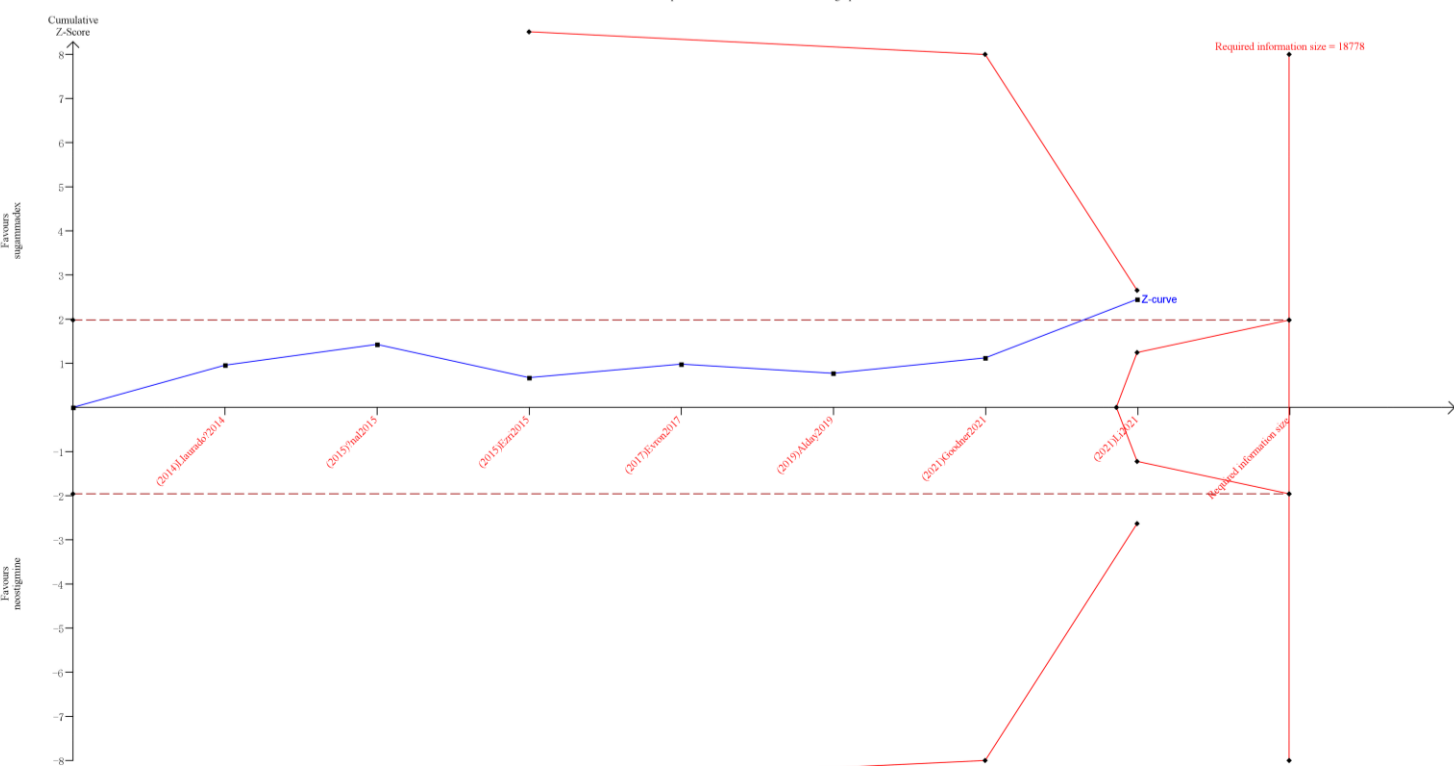

Supplement: Supplementary file 5 — Additional file 5: Supplementary file 5. TSA. [file 12871_2023_2094_MOESM5_ESM.pdf]
