# Supplementary material for: Postoperative pulmonary complications after sugammadex reversal of neuromuscular blockade: a systematic review and meta-analysis with trial sequential analysis
Source: BMC Anesthesiol. 2023 Apr 20;23:130. doi: 10.1186/s12871-023-02094-0 (PMC10116764; doi:10.1186/s12871-023-02094-0)
Supplement: Supplementary file 4 — Additional file 4: Supplementary file 4. Quality of evidence by GRADE. [file 12871_2023_2094_MOESM4_ESM.docx]

**Supplementary file 4: GRADE summary of primary outcomes**

**Sugammadex compared to neostigmine for PPCs**

| **Certainty assessment** | | | | | | | **№ of patients** | | **Effect** | | **Certainty** | **Importance** |
| --- | --- | --- | --- | --- | --- | --- | --- | --- | --- | --- | --- | --- |
| **№ of studies** | **Study design** | **Risk of bias** | **Inconsistency** | **Indirectness** | **Imprecision** | **Other considerations** | **sugammadex** | **neostigmine for PPCs** | **Relative (95% CI)** | **Absolute (95% CI)** |  |  |
| **desaturation** | | | | | | | | | | | | |
| 8 RCTs + 5 OSs | | serious^a,b,c,d,e^ | serious^f^ | not serious | not serious | none | 2255/5221 (43.2%) | 2130/4732 (45.0%) | **RR 0.82** (0.63 to 1.05) | **81 fewer per 1,000** (from 167 fewer to 23 more) | ⨁⨁◯◯ Low |  |
| **pneumonia** | | | | | | | | | | | | |
| 5 RCTs + 5 OSs | | serious^a,c,e,g^ | not serious | not serious | not serious | publication bias strongly suspected^h^ | 368/26938 (1.4%) | 782/31951 (2.4%) | **RR 0.65** (0.49 to 0.85) | **9 fewer per 1,000** (from 12 fewer to 4 fewer) | ⨁⨁◯◯ Low |  |
| **atelectasis** | | | | | | | | | | | | |
| 4 RCTs + 3 OSs | | serious^c,g^ | serious^i,j^ | not serious | not serious | publication bias strongly suspected^h^ | 307/1248 (24.6%) | 380/1251 (30.4%) | **RR 0.64** (0.42 to 0.98) | **109 fewer per 1,000** (from 176 fewer to 6 fewer) | ⨁◯◯◯ Very low |  |
| **NIV** | | | | | | | | | | | | |
| 2 RCTs + 3 OSs | | serious^a,d,k^ | serious^i,j^ | not serious | not serious | publication bias strongly suspected^h^ | 370/26963 (1.4%) | 615/26444 (2.3%) | **RR 0.65** (0.43 to 0.98) | **8 fewer per 1,000** (from 13 fewer to 0 fewer) | ⨁◯◯◯ Very low |  |
| **reintubation** | | | | | | | | | | | | |
| 5 RCTs + 4 OSs | | serious^a,b,d,e^ | not serious | not serious | very serious^l^ | publication bias strongly suspected^h^ | 33/3342 (1.0%) | 139/8437 (1.6%) | **RR 0.62** (0.43 to 0.91) | **6 fewer per 1,000** (from 9 fewer to 1 fewer) | ⨁◯◯◯ Very low |  |

**OS:** observational study; **RCT:** randomized controlled trial; **CI:** confidence interval; **RR:** risk ratio

#### Explanations

a. Lack of blinding, random sequence generation and allocation concealment in Alday 2019 study

b. Lack of blinding and allocation concealment in Evron 2017 study

c. Lack of blinding in Togioka 2020 study

d. Lack of blinding in Ünal 2015 study

e. Use of inappropriate controls (use of no reversal agent or neostigmine as control group in Ledowski 2013 study, or outcome data extracted from unmatched cohort in Li 2021 study)

f. The definition of desaturation varied widely across studies

g. Lack of allocation concealment in Çitil 2019 study

h. The publication bias was not assessed because of the limit of the amount of included studies, and which strongly suspected all plausible residual confounding would reduce the demonstrated effect

i. CIs showed minimal or no overlap

j. I2 values indicated moderate or high thresholds for statistical heterogeneity

k. No information about adequately adjusting for prognostic imbalance in Ezri 2015 study

l. Total number of events were less than 300
